# Exploring Electrode Placements to Optimize the Identification and Measurement of Early Auditory Evoked Potentials

**DOI:** 10.3390/audiolres13060085

**Published:** 2023-12-11

**Authors:** Kailyn A. McFarlane, Jason Tait Sanchez

**Affiliations:** 1 Roxelyn and Richard Pepper Department of Communication Sciences and Disorders, Northwestern University, Evanston, IL 60208, USA; jason.sanchez@northwestern.edu; 2 Knowles Hearing Center, Northwestern University, Evanston, IL 60208, USA; 3 Department of Neurobiology, Northwestern University, Evanston, IL 60208, USA

**Keywords:** electrocochleography, compound action potential, auditory brainstem response, auditory evoked potentials, electrode placement, cochlear synaptopathy

## Abstract

Cochlear synaptic loss (termed cochlear synaptopathy) has been suggested to contribute to suprathreshold hearing difficulties. However, its existence and putative effects in humans remain inconclusive, largely due to the heterogeneous methods used across studies to indirectly evaluate the health of cochlear synapses. There is a need to standardize proxies of cochlear synaptopathy to appropriately compare and interpret findings across studies. Early auditory evoked potentials (AEPs), including the compound action potential (AP)/Wave I of the auditory brainstem response are a popular proxy, yet remain variable based on technical considerations. This study evaluated one such consideration—electrode array (i.e., montage)—to optimize the use of early AEP waveforms. In 35 young adults, electrocochleography (ECochG) responses were collected using vertical and horizontal montages. Standard ECochG measures and AP/Wave I and Wave II peak-to-trough amplitudes and latencies were compared between montages. Vertical montage recordings consistently produced significantly larger AP/Wave I peak-to-trough amplitudes compared to horizontal recordings. These findings support the use of a vertical electrode montage for optimal recordings of peripheral cochlear nerve activity. As cochlear synaptopathy continues to be explored in humans, the methods highlighted here should be considered in the development of a standardized assessment.

## 1. Introduction

Cochlear synaptopathy (CS), or the loss of cochlear synapses, has been identified in animal models due to aging and excessive noise exposure [1,2]. The synaptic loss between inner hair cells (IHCs) and spiral ganglions neurons (SGNs) can occur separately from outer hair cell loss and permanent threshold shifts [3]. This discovery prompted the investigation of possible CS in humans, particularly in individuals with clinically normal audiometric thresholds who report auditory complaints including tinnitus, hyperacusis, and difficulty with speech perception in noisy environments [3,4]. However, the health of cochlear synapses in humans must be assessed non-invasively and indirectly. Prior work has employed a variety of methods to accomplish this non-invasive assessment, including subjective questionnaires and audiometric and electrophysiologic measures (see Le Prell [5] and DiNino et al. [6] for comprehensive reviews). However, these measures have produced mixed results, making it difficult to interpret whether CS indeed exists in humans and to what extent it contributes to auditory deficits. Thus, standardized proxies for CS are needed to adequately compare and interpret findings across studies.

In animal models, CS is often first identified using electrophysiological measures such as the auditory brainstem response (ABR), and then validated through post-mortem histological counts of IHC-SGN synapses [1]. While we currently lack non-invasive means to directly quantify these synapses in living humans, early auditory evoked potentials (AEPs) such as the ABR are a well-established tool in rodent models and humans alike for assessing neural integrity in these anatomical structures. A measure of particular value is Wave I of the ABR, which represents the summed postsynaptic neural activity at the peripheral cochlear nerve [7]. Studies in rodent models have used Wave I of the ABR to assess noise-induced and age-related damage to the peripheral cochlear nerve, showing a permanent reduction in Wave I peak-to-trough amplitude despite only temporary shifts in behavioral thresholds [1,2,8,9]. This evidence suggests that Wave I of the ABR could be a promising non-invasive method of detecting CS in humans [10].

However, Wave I of the ABR has demonstrated a high variability both between and within individuals [11,12]. This variability comes from patient and methodological factors [13,14] and likely contributes to the mixed results observed across human studies that use Wave I as a proxy for CS. Moreover, the ABR’s function to broadly capture the entire brainstem’s response (cochlear nerve to inferior colliculus [15,16]) may result in less sensitivity to subtle changes in the peripheral cochlear nerve. A more targeted method of recording the summed neural response of the peripheral cochlear nerve is electrocochleography (ECochG), which records electrical activity from within and around the cochlea [17]. In ECochG recordings, the activity originating from the peripheral cochlear nerve is referred to as the compound action potential (AP); synonymous with Wave I of the ABR. This activity will henceforth be referred to as AP/Wave I in this paper.

While ABR and ECochG are often lumped together in CS literature, there are key differences in their standard recording parameters that make ECochG a more ideal method for measuring AP/Wave I (see Harris and Bao [4] for review). For example, the recording electrode in ECochG is placed inside the ear canal or against the tympanic membrane rather than on the mastoid or earlobe as in ABRs [18]. Moving the electrode closer to the source of neural generation produces a more robust and reliable measure of AP/Wave I [19]. Another key difference—and the focus of this study—is the electrode array, or montage, used.

**Figure 1:**
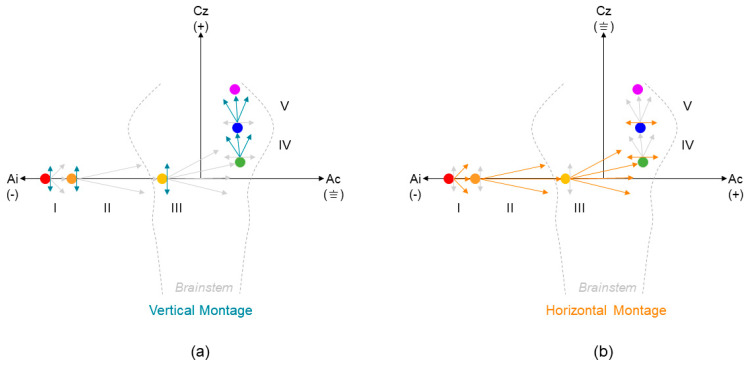
Dipole Theory. Schematic of stationary dipole spread along the auditory brainstem (adopted from Scherg and von Cramon 1985 [21]). (**a**) Blue arrows indicate propagation of activity in the vertical plane, optimally captured in a vertical electrode montage. (**b**) Orange arrows indicate propagation of activity in horizontal plane, optimally captured in a horizontal electrode montage. Gray arrows indicate less emphasis on dipole spread in both montage configurations. Note that only predominant ipsilateral/contralateral contributions are shown for simplicity. In ascending order, indicated by warm to cool colored dots, dipole activity originating from Waves: (I) Distal Cochlear Nerve, (II) Proximal Cochlear Nerve, (III) Cochlear Nucleus, (IV) Superior Olivary Complex, (V) Lateral Lemniscus into Inferior Colliculus. Ai = ipsilateral ear; Ac = contralateral ear; Cz = midline, top of head. (−) = inverting electrode; (+) = non-inverting electrode; (⏚) = common ground electrode. Gray dashed outline in A and B indicates brainstem from medulla to midbrain.

Traditionally, ECochGs are recorded using a horizontal montage, where the inverting and non-inverting electrodes are positioned in the horizontal plane (i.e., opposite ears), while ABRs use a vertical montage (e.g., ipsilateral ear to vertex) [20]. This is carried out with the purpose of emphasizing different components of early AEP waveforms based on the goals of testing (e.g., monitoring endolymphatic hydrops versus estimating thresholds). According to the spatio-temporal dipole model [21], the propagation of a dipole’s local electrical potential to a surface electrode is dependent both on the position of the electrodes and the anatomical orientation of the dipole location (Figure 1). That is, a dipole with a predominantly horizontal orientation would be more completely captured by electrodes positioned in the horizontal plane rather than the vertical plane. The first dipole in the early AEP waveform originates in the distal portion of the cochlear nerve, making up the AP/Wave I. Due to the horizontal orientation of cochlear nerve fiber tracks in the adult human skull (i.e., within the internal auditory canal), we hypothesize that electrodes differentially recording in a horizontal plane optimally detect the summed activity originating from the dipole generated by the peripheral cochlear nerve.

However, there is limited literature testing or supporting this idea in practice. One study performed over four decades ago showed little to no differences in Wave I between horizontal (mastoid-to-mastoid) and vertical (mastoid-to-forehead) montages [22]. In fact, the only difference observed was a small reduction in Wave I amplitude in the horizontal montage. Thus, the current study aims to add to the paucity of literature regarding montage effects on AP/Wave I peak-to-trough amplitude. By doing so, we aim to advance the search for a rigorous and reliable non-invasive measure of summed neural activity at the peripheral cochlear nerve.

## 2. Materials and Methods

Thirty-five young adults (4 male) between the ages 20 and 29 (mean = 23.14 years) participated in the study. All participants passed otoscopy and a pure tone audiometry screening to demonstrate normal hearing thresholds (≤20 dB HL at 0.25, 0.5, 1, 2, 3, 4, 6, 8 kHz). Participants were informed of the nature of the experiment and participant consent was obtained from each individual. All experimental procedures were approved by the Northwestern University Institutional Review Board (STU00215672). In total, 37 individuals participated, but 2 were excluded from analysis due to excessively noisy Electrocochleography (ECochG) recordings that prohibited the identification of waveform components.

ECochGs were evoked using a 100 μs broadband click, in alternating polarity, generated by the Intelligent Hearing Systems (IHS; Miami, FL, USA) Smart EP Duet platform. The stimulus was presented to the right ear at a click rate of 9.1/s at 90 dB nHL (94 dB SPL RMS) through a gold-foil TIPtrode attached to an ER-3C insert earphone (Etymotic Research, Elk Grove Village, IL, USA). The non-test ear was unobstructed. Evoked responses were processed online through the IHS Smart EP Duet platform. Signals were amplified with a gain of 100,000 and band-pass filtered between 10 and 1500 Hz. Data were collected over a 12.8 ms epoch at a 40 kHz sample rate and averaged for a minimum of 1024 repetitions. A minimum of two repeatable waveforms were recorded and then added to obtain a grand averaged response for each montage (2048 sweeps).

**Figure 2:**
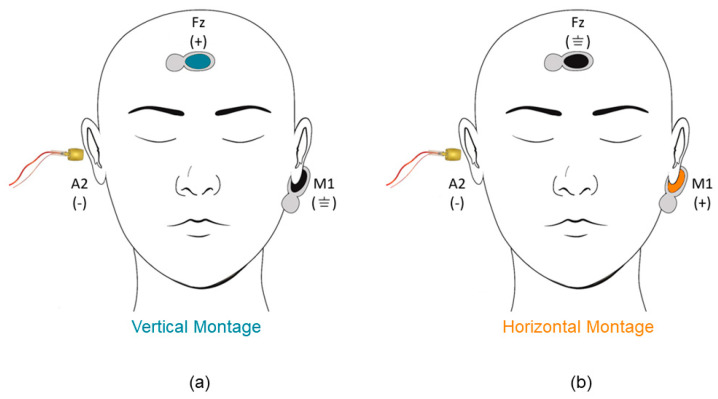
Montage Configuration. Illustration of electrode placements for the (**a**) vertical and (**b**) horizontal montage. A2 = right (ipsilateral) ear canal; M1 = left (contralateral) mastoid; Fz = high center forehead. (−) = inverting; (+) = non-inverting; (⏚) = common ground.

Electrode sites were prepped with alcohol and NUPrep™ skin prepping gel (Weaver and Company; Aurora, CO, USA). Two disposable snap surface electrodes (Ambu Neuroline surface electrodes; Ambu INC., Columbia, MD, USA) were placed onto the high center forehead (Fz) and contralateral mastoid (M1). A commercially available ear canal electrode (TIPtrode Etymotic Research, Elk Grove Village, IL, USA) was placed in the ipsilateral ear canal (A2) securely and as deep as possible. Vertical and horizontal single-channel montages were used for data collection. Both montages used the TIPtrode as the inverting electrode. The non-inverting electrode was placed on Fz for the vertical montage and M1 for the horizontal montage. The remaining electrode in each respective montage was used as common ground. Figure 2 is a schematic showing the electrode placement for the vertical and horizontal montages.

Data collection took place in a sound-treated room. Participants were seated in a reclined chair and prepped for electrode placement. Once all electrodes were placed, impedance was confirmed to be ≤5 kΩ with an inter-electrode impedance of ≤3 kΩ. Room lights were turned off during collection. The order in which montage recordings were obtained was randomized. The grand average waveform of the two responses recorded for each montage were used to characterize the participant’s response.

Waveform components were marked in the IHS SmartEP software (version 5.41.01) using visual overlay cursors. All components were initially identified and marked by the first author during data collection and later confirmed by the senior author, who is a state licensed and certified audiologist. Any inter-scorer disagreements between the two judges were settled through reviewing the data together. Three standard ECochG components were marked on each waveform: baseline, the summating potential (SP) peak, the compound action potential (AP) peak/Wave I. Baseline was identified at the flattest point within the first millisecond from stimulus onset. Latency of the SP was defined as the positive peak on the ascending shoulder of the AP. For the primary measures of the study, the trough following the AP/Wave I peak was also marked, as well as the peak and following trough for Wave II. Standard ECochG measures and AP/Wave I and Wave II peak-to-trough amplitudes and latencies were compared across the two montages using paired *t*-tests. Statistical significance was defined as *p* < 0.05. All statistical analysis was performed using GraphPad Prism version 8.0.2 for Windows (GraphPad Software, La Jolla, CA, USA).

## 3. Results

### 3.1. Electrode Montage Effects on Standard Measures of Electrocochleography (ECochG)

**Figure 3:**
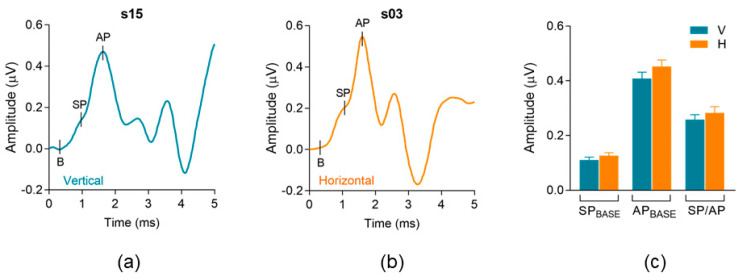
Standard ECochG waveform components. (**a**,**b**) show representative ECochG traces from two participants in the vertical (blue) and horizontal (orange) montage, respectively. Each trace is an average of two repeatable recordings, resulting in an average of 2048 sweeps. Standard ECochG components are labeled; B = baseline, SP = summating potential, AP = compound action potential. (**c**) Comparison of standard ECochG measures between montages.

Figure 3a,b shows ECochG waveforms recorded with vertical and horizontal electrode montages from two different randomly selected participants. The recordings show standard ECochG morphology and their marked waveform components: baseline (B), summating potential (SP), and compound action potential (AP). Figure 3c shows standard ECochG measures—SP and AP amplitudes relative to baseline and SP/AP ratio—compared across vertical and horizontal montages. All three measures were not significantly different between electrode montages. SP amplitude (re: baseline, SP_BASE_) was not significantly different (*p* = 0.25) between the vertical (µ = 0.11 µV) and horizontal (µ = 0.12 µV) montages. AP amplitude (re: baseline, AP_BASE_) was not significantly different (*p* = 0.09) between vertical (µ = 0.41 µV) and horizontal (µ = 0.45 µV) montages. Lastly, SP/AP amplitude ratio was not significantly different (*p* = 0.35) between vertical (µ = 0.25 µV) and horizontal (µ = 0.28 µV) montages. Table 1 includes the quantitative analysis and descriptive statistics for each standard ECochG component analyzed between the vertical and horizontal montages.

### 3.2. Electrode Montage Effects on Measures of Early Auditory Evoked Activity

#### 3.2.1. Variability and Trends within Subjects and between Montages

**Figure 4:**
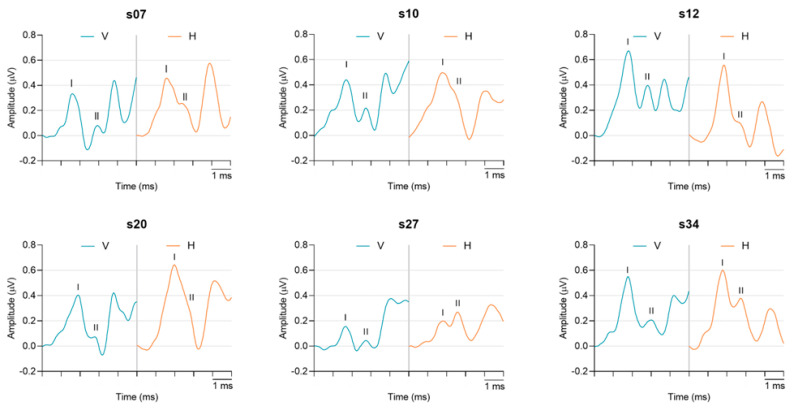
Within subject variability between montages. Six randomly selected participants’ ECochG recordings in vertical (blue) and horizontal (orange) montages. Each trace is an average of two repeatable recordings (2048 total averaged sweeps). AP/Wave I and Wave II peaks are labeled with the corresponding roman numeral.

**Figure 5:**
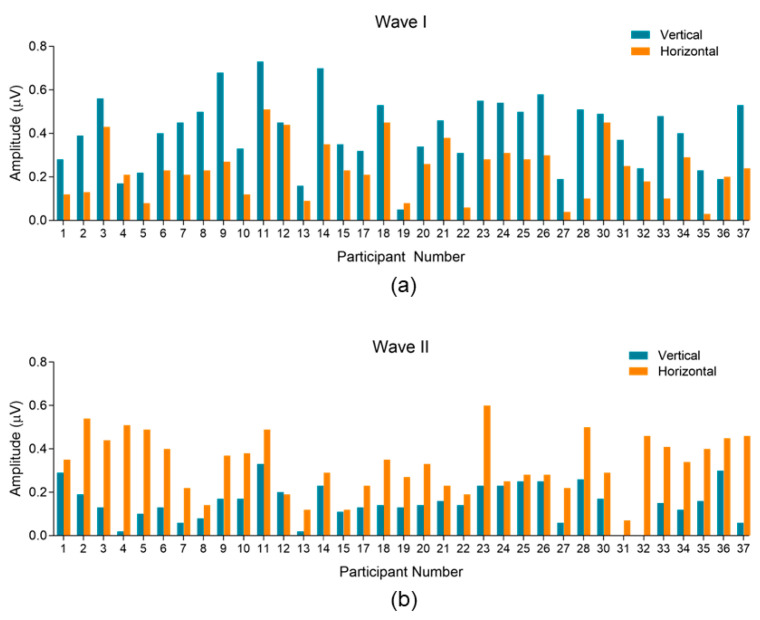
Peak-to-trough amplitude trends between montages. (**a**) AP/Wave I and (**b**) Wave II peak-to-trough amplitudes in both montages for each participant. Amplitudes from the vertical montage are blue, horizontal are orange. Participants #16 and #29 were omitted from analysis due to excessive noise in their recordings that resulted in failure to identify all waveform components. Final participants included *n* = 35.

Figure 4 shows ECochG waveforms recorded with vertical and horizontal electrode montages for six randomly selected participants. The recordings are representative of within-subject waveform variability across the population sampled. For this and subsequent figures, standard ECochG waveform components (i.e., SP_BASE_, AP_BASE_, and SP/AP ratio) are not labeled. Rather, the peak-to-trough amplitudes of AP/Waves I and II are labeled and characterized. Waveforms for all 35 participants can be found in Supplemental Figure S1. The peak-to-trough amplitudes of Wave I and Wave II in each participant are shown for both montages in Figure 5. In general, the AP/Wave I peak-to-trough amplitude was larger in the vertical montage recordings (a, top panel) while Wave II was larger in the horizontal montage recordings (b, bottom panel). These montage-specific trends showed minimal variability across participants. Specifically, 91% (32/35) of participants had larger AP/Wave I peak-to-trough amplitudes in the vertical montage, while 97% (34/35) had larger Wave II peak-to-trough amplitudes in the horizontal montage.

#### 3.2.2. Waveform Peak-to-Trough Amplitude and Peak Latency

**Figure 6:**
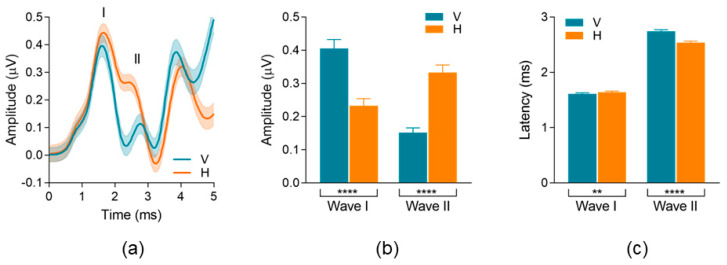
Population waveform differences between montages. (**a**) Grand average waveforms (*n* = 35) for vertical (blue) and horizontal (orange) montages. Bold line = grand average; Shading = SEM. (**b**) Comparison of AP/Wave I and Wave II peak-to-trough amplitudes between montages. (**c**) Comparison of AP/Wave I and Wave II latency between montages. Error bars = SEM. ** denotes *p* < 0.01, **** denotes *p* < 0.0001.

Figure 6a shows population grand averaged ECochG waveform recordings (±1 SEM, *n* = 35) for the vertical montage (blue trace) and the horizontal montage (orange trace). Like the individual data highlighted in Figure 4, there are clear differences in ECochG morphology between the vertical and horizontal montage recordings, encompassing both AP/Waves I and II. Table 2 includes the quantitative analysis and descriptive statistics for AP/Wave I and Wave II peak-to-trough amplitude and latency parameters between montages.

AP/Wave I and Wave II peak-to-trough amplitude comparisons are shown in Figure 6b. Wave I peak-to-trough amplitude was significantly larger (*p* < 0.0001) in the vertical montage (µ = 0.40 µV) compared to the horizontal montage (µ = 0.23 µV). This results in an average AP/Wave I peak-to-trough amplitude increase of 74% when using a vertical montage. Conversely, Wave II peak-to-trough amplitude was significantly larger (*p* < 0.0001) in the horizontal montage (µ = 0.33 µV) compared to the vertical montage (µ = 0.15 µV). This results in an average Wave II peak-to-trough amplitude increase of 120% when using a horizontal montage. AP/Wave I and II peak latency comparison is shown in Figure 6c, and descriptive values are listed in Table 2. Wave I peak latency was slightly earlier—albeit significant (*p* = 0.002)—in the vertical montage (µ = 1.61 ms) compared to the horizontal montage (µ = 1.64 ms). Conversely, Wave II peak latency was significantly earlier (*p* < 0.0001) in the horizontal montage (µ = 2.54 ms) compared to the vertical montage (µ = 2.74 ms).

### 3.3. Residual Noise Analysis

To determine if other internal factors (e.g., physiologic noise) contributed to the differences observed in peak-to-trough amplitudes of early auditory evoked potentials between montages, we analyzed the residual noise of each ECochG recording. This calculation is performed automatically in the IHS SmartEP software and is described in detail in Keesling et al. [23]. We found that residual noise was not significantly different (*p* = 0.5) between the vertical (µ = 0.45 µV) and horizontal (µ = 0.49 µV) montages.

## 4. Discussion

The purpose of this study was to compare early auditory evoked potential (AEP) waveform components between vertical and horizontal electrode arrays, or montages. This was conducted to further identify optimal recording techniques of the summed activity at the cochlear nerve synapse, which is a leading proxy in the investigation of cochlear synaptopathy (CS) in humans. While there were no differences in standard ECochG measurements between montages, we observed significant differences in peak-to-trough amplitude measurements for both AP/Wave I and Wave II. Interestingly, these amplitude trends behaved in opposite directions: where AP/Wave I amplitude was largest in the vertical montage, and Wave II amplitude was largest in the horizontal montage.

### 4.1. Standard Electrocochleography (ECochG) Measures

The standard measures in ECochG recordings include baseline-to-peak amplitude of the summating potential (SP), compound action potential (AP), and the ratio of the two (i.e., SP/AP ratio). In this study, no differences were found in these measures between vertical and horizontal montage recordings. This was somewhat surprising given the well-established practice of recording ECochGs using a horizontal montage for the purpose of optimizing the identification and measurement of these waveform components [22]. A possible explanation could lie in the sheer proximity of the sites of electric potential to the inverting electrode. The summating potential—generated by the direct current from inner hair cells (IHCs) [24]—and the peak of AP/Wave I may be maximally recorded in both horizontal and vertical montages because the inverting electrode is close enough to capture the entirety of the voltage propagation from these anatomical sources, making the position of the non-inverting electrode irrelevant. This would be distinct from the successive, deeper-positioned dipoles measured in early AEPs, where capturing the full extent of their responses is reliant on the differential recording of activity between the inverting and non-inverting electrodes.

A few studies investigating CS in humans have analyzed standard ECochG measures, specifically SP/AP amplitude ratios [10,25]. If such measures are to continue to be used, the results of this study suggest either a vertical or horizontal montage could be employed. However, it should be noted that these findings should only be applied to ECochG recordings using an alternating stimulus polarity. While an alternating polarity is the standard choice for limiting stimulus artifact and eliminating the cochlear microphonic, it can obscure certain differences in SP and AP that arise when recording in rarefaction and condensation polarities [24]. Future work should investigate whether the observed montage differences (or lack thereof) in standard ECochG measures are maintained when recording with rarefaction and condensation polarities.

### 4.2. Montage Differences in Early AEPs: Waves I and II Peak-to-Trough Amplitudes

Contrary to our hypothesis based on the spatio-temporal dipole model, electrodes differentially recording in a horizontal plane did not optimally detect the summed activity originating from the dipole generated by the peripheral cochlear nerve. This was strongly observed in our data; 91% (32/35) of participants showed a larger AP/Wave I peak-to-trough amplitude in the vertical montage. On average, recording in the vertical montage resulted in a 74% increase in AP/Wave I peak-to-trough amplitude. We believe this result to be closely related to what is observed in Wave II morphology between montages. In the vertical montage, Wave II occurs significantly later in time and has a significantly reduced peak-to-trough amplitude when compared to horizontal montage recordings. This trend is also highly consistent across participants, with 97% (34/35) showing larger Wave II peak-to-trough amplitudes in the horizontal montage compared to the vertical montage. This delayed onset of Wave II in the vertical montage—supported by earlier experiments [26]—as well as its diminished presence, explains why a larger AP/Wave I peak-to-trough amplitude is observed in the vertical montage. That is, the earlier Wave II peak latency in the horizontal montage impedes, or cuts short, the conclusion of AP/Wave I’s trough, resulting in a reduced peak-to-trough amplitude.

While these results were unexpected, they are not necessarily in conflict with the spatio-temporal dipole model. Wave II represents the stationary dipole originating from the proximal cochlear nerve and its output to the ipsilateral cochlear nucleus [27]. This dipole represents transverse propagation along auditory nerve fibers in the internal auditory canal as they transition into the posterior fossa [21]. As such, it is not surprising that Wave II is emphasized in the horizontal montage over the vertical montage, and that the more optimal recording of this activity could interfere with capturing the entirety of AP/Wave I activity.

Most human studies investigating CS that use early AEPs have specifically chosen peak-to-trough amplitude of AP/Wave I as their method of indirectly surveying the health of cochlear synapses [28]. As such, establishing a robust and reliable method of obtaining this measure is critical. In this study, we have demonstrated consistently larger recordings of AP/Wave I peak-to-trough amplitude in the vertical montage compared to the horizontal montage, suggesting the vertical montage is a more optimal choice for this measure. Furthermore, using a vertical montage to optimize AP/Wave I waveform characteristics has the potential to improve subjective (e.g., visual) waveform identification (prone to human error) as well as automated quantification approaches, further minimizing variability in the data [4].

### 4.3. Variability Related to Electrode Sites and Types

It is important to acknowledge that the inverting electrode used for this study’s recordings was an ear canal electrode (TIPtrode) rather than a tympanic membrane (TM) electrode, another popular option for ECochG recordings. While TIPtrodes have been shown to produce smaller amplitudes and higher variability [29] than TM electrodes, the current study chose to use the TIPtrode due to its ease of use, patient comfortability, and familiarity with audiologists (including personnel involved in data collection). It should be noted that, with proper training, similar levels of patient comfort and clinician efficiency can be achieved with TM electrodes, and they should remain at the top of the options under consideration when recording ECochGs in clinical and research settings alike. Importantly, the quantitative differences between TM electrodes and ear canal electrodes are observed only for standard ECochG measures including SP and SP/AP ratio [29]. How variability differs between these electrodes for peak-to-trough amplitudes of Wave I and Wave II has been less reported on. It has been reported, however, that SP-related measures are generally less reliable than measures of Wave I [19]. Furthermore, despite the variability associated with Wave I measurements, it has been shown that, overall, ear canal electrodes produce larger Wave I amplitudes and lower variability compared to mastoid or earlobe electrodes [19,30]. All things considered, the TIPtrode proves itself to be a viable option for the application of measuring the summed activity of the peripheral cochlear nerve (i.e., Wave I peak-to-trough amplitude). The results and recommendations of this study should not be applied to AEP recordings using inverting electrode types and locations other than the TIPtrode/ear canal.

Another possible explanation for these results is the difference in physiologic noise that may be present between montages. Since the non-inverting electrode is placed on the contralateral mastoid in the horizontal montage, it is important to rule out any influence from myogenic noise caused by the post-auricular muscle that overlays the mastoid. To explore this, we compared the residual noise values from each ECochG recording between montages. No difference was found, suggesting the main results of this study were not influenced by such factors.

## 5. Conclusions

As cochlear synaptopathy continues to be investigated in humans, there is a distinct need for a standardized non-invasive measure to be used across studies. Our study focused on optimizing electrocochleography, an early AEP that measures the summed neural activity at the peripheral cochlear nerve, through electrode placement. Our findings show that vertical montage recordings provide a more robust measure of AP/Wave I peak-to-trough amplitude compared to horizontal montage recordings. Therefore, we recommend recording in a vertical montage for a more sensitive measure of the summed activity at the peripheral cochlear nerve.

## Figures and Tables

**Table 1:** Descriptive statistics and statistical analysis summary across standard ECochG waveform components and montages.

	Montage	Median	Mean (SD)	Min–Max
SP_BASE_ *p* = 0.25	Vertical	0.10	0.11 (0.06)	0.01–0.27
	Horizontal	0.13	0.12 (0.06)	0.02–0.27
AP_BASE_ *p* = 0.09	Vertical	0.42	0.41 (0.14)	0.17–0.68
	Horizontal	0.50	0.45 (0.13)	0.21–0.67
SP/AP Ratio *p* = 0.35	Vertical	0.24	0.25 (0.10)	0.06–0.55
	Horizontal	0.30	0.28 (0.13)	0.04–0.69

**Table 2:** Descriptive statistics and statistical analysis summary across early auditory evoked potential waveform components and montages.

	Wave	Montage	Median	Mean (SD)	Min–Max
Peak-to-Trough Amplitude (μV)	AP/Wave I *p* < 0.0001	Vertical	0.40	0.40 (0.16)	0.05–0.73
		Horizontal	0.23	0.23 (0.13)	0.03–0.51
	Wave II *p* < 0.0001	Vertical	0.14	0.15 (0.08)	0.00–0.33
		Horizontal	0.34	0.33 (0.13)	0.07–0.60
Peak Latency (ms)	AP/Wave I *p* = 0.002	Vertical	1.63	1.61 (0.10)	1.35–1.90
		Horizontal	1.65	1.64 (0.13)	1.35–1.98
	Wave II *p* < 0.0001	Vertical	2.77	2.74 (0.18)	2.25–3.03
		Horizontal	2.58	2.54 (0.16)	2.23–2.85

## Data Availability

The data presented in this study are available on request from the corresponding author.

## Supplementary Materials

The following supporting information can be downloaded at: https://www.mdpi.com/article/10.3390/audiolres13060085/s1, Figure S1: Vertical and horizontal ECochG traces for all participants.

## References

[1] Kujawa S.G., Liberman M.C. (2009). Adding Insult to Injury: Cochlear Nerve Degeneration after “Temporary” Noise-Induced Hearing Loss. J. Neurosci..

[2] Sergeyenko Y., Lall K., Liberman M.C., Kujawa S.G. (2013). Age-Related Cochlear Synaptopathy: An Early-Onset Contributor to Auditory Functional Decline. J. Neurosci..

[3] Kujawa S.G., Liberman M.C. (2015). Synaptopathy in the noise-exposed and aging cochlea: Primary neural degeneration in acquired sensorineural hearing loss. Hear. Res..

[4] Harris K.C., Bao J. (2022). Optimizing non-invasive functional markers for cochlear deafferentation based on electrocochleography and auditory brainstem responses. J. Acoust. Soc. Am..

[5] Le Prell C.G. (2019). Effects of noise exposure on auditory brainstem response and speech-in-noise tasks: A review of the literature. Int. J. Audiol..

[6] DiNino M., Holt L.L., Shinn-Cunningham B.G. (2022). Cutting Through the Noise: Noise-Induced Cochlear Synaptopathy and Individual Differences in Speech Understanding Among Listeners with Normal Audiograms. Ear Hear..

[7] Møller A.R., Jannetta P.J. (1981). Compound action potentials recorded intracranially from the auditory nerve in man. Exp. Neurol..

[8] Furman A.C., Kujawa S.G., Liberman M.C. (2013). Noise-induced cochlear neuropathy is selective for fibers with low spontaneous rates. J. Neurophysiol..

[9] Lobarinas E., Spankovich C., Le Prell C.G. (2017). Evidence of “hidden hearing loss” following noise exposures that produce robust TTS and ABR wave-I amplitude reductions. Hear. Res..

[10] Liberman M.C., Epstein M.J., Cleveland S.S., Wang H., Maison S.F. (2016). Toward a Differential Diagnosis of Hidden Hearing Loss in Humans. PLoS ONE.

[11] Beattie R.C. (1988). Interaction of click polarity, stimulus level, and repetition rate on the auditory brainstem response. Scand. Audiol..

[12] Lauter J.L., Loomis R.L. (1988). Individual differences in auditory electric responses: Comparisons of between-subject and within-subject variability. II. Amplitude of brainstem Vertex-positive peaks. Scand. Audiol..

[13] Stockard J.E., Stockard J.J., Westmoreland B.F., Corfits J.L. (1979). Brainstem Auditory-Evoked Responses: Normal Variation as a Function of Stimulus and Subject Characteristics. Arch. Neurol..

[14] Plack C.J., Léger A., Prendergast G., Kluk K., Guest H., Munro K.J. (2016). Toward a Diagnostic Test for Hidden Hearing Loss. Trends Hear..

[15] Hashimoto I., Ishiyama Y., Yoshimoto T., Nemoto S. (1981). Brain-Stem Auditory-Evoked Potentials Recorded Directly from Human Brain-Stem and Thalamus. Brain.

[16] Møller A.R., Jho H.D., Yokota M., Jannetta P.J. (1995). Contribution from crossed and uncrossed brainstem structures to the brainstem auditory evoked potentials: A study in humans. Laryngoscope.

[17] Ferraro J.A., Krishnan G. (1997). Cochlear Potentials in Clinical Audiology. Audiol. Neurotol..

[18] Simpson M.J., Jennings S.G., Margolis R.H. (2020). Techniques for Obtaining High-quality Recordings in Electrocochleography. Front. Syst. Neurosci..

[19] Prendergast G., Tu W., Guest H., Millman R.E., Kluk K., Couth S., Munro K.J., Plack C.J. (2018). Supra-threshold auditory brainstem response amplitudes in humans: Test-retest reliability, electrode montage and noise exposure. Hear. Res..

[20] Singh C.B., Mason S.M. (1981). Simultaneous recording of extra-tympanic electrocochleography and brainstem evoked responses in clinical practice. J. Laryngol. Amp Otol..

[21] Scherg M., von Cramon D. (1985). A new interpretation of the generators of BAEP waves I-V: Results of a spatio-temporal dipole model. Electroencephalogr. Clin. Neurophysiol..

[22] Ruth R.A., Hildebrand D.L., Cantrell R.W. (1982). A Study of Methods Used to Enhance Wave I in the Auditory Brain Stem Response. Otolaryngol. Head. Neck Surg..

[23] Keesling D.A., Parker J.P., Sanchez J.T. (2017). A Comparison of Commercially Available Auditory Brainstem Response Stimuli at a Neurodiagnostic Intensity Level. Audiol. Res..

[24] Ferraro J.A., Durrant J.D. (2006). Electrocochleography in the evaluation of patients with Ménière’s disease/endolymphatic hydrops. J. Am. Acad. Audiol..

[25] Grant K.J., Mepani A.M., Wu P., Hancock K.E., de Gruttola V., Liberman M.C., Maison S.F. (2020). Electrophysiological markers of cochlear function correlate with hearing-in-noise performance among audiometrically normal subjects. J. Neurophysiol..

[26] Ananthanarayan A.K., Durrant J.D. (1991). On the Origin of Wave II of the Auditory Brain Stem Evoked Response. Ear Hear..

[27] Møller A.R., Jannetta P., Bennett M., Møller M.B. (1981). Intracranially recorded responses from the human auditory nerve: New insights into the origin of brain stem evoked potentials (BSEPs). Electroencephalogr. Clin. Neurophysiol..

[28] Bramhall N.F. (2021). Use of the auditory brainstem response for assessment of cochlear synaptopathy in humans. J. Acoust. Soc. Am..

[29] Lefler S.M., Kaf W.A., Ferraro J.A. (2021). Comparing Simultaneous Electrocochleography and Auditory Brainstem Response Measurements Using Three Different Extratympanic Electrodes. J. Am. Acad. Audiol..

[30] Ferraro J.A. (2010). Electrocochleography: A Review of Recording Approaches, Clinical Applications, and New Findings in Adults and Children. J. Am. Acad. Audiol..

